# Where Evidence‐Based Medicine Meets AI: Promise, Pitfalls, and Practice

**DOI:** 10.1002/aet2.70210

**Published:** 2026-06-21

**Authors:** Sangil Lee, Joshua Davis, Ken Milne, Christina Shenvi, Lars K. Beattie, Martin Wegman, Laura Melville, Richard D. Shih, Bryan Kane

**Affiliations:** ^1^ Department of Emergency Medicine University of Iowa Carver College of Medicine Iowa City Iowa USA; ^2^ Vituity Kansas USA; ^3^ Department of Emergency Medicine Strathroy Middlesex General Hospital Strathroy Ontario Canada; ^4^ Department of Emergency Medicine University of North Carolina at Chapel Hill Chapel Hill North Carolina USA; ^5^ Department of Emergency Medicine University of Florida College of Medicine Gainesville Florida USA; ^6^ Orange Park Emergency Medicine Orange Park Florida USA; ^7^ Department of Emergency Medicine NewYork‐Presbyterian Brooklyn Methodist Hospital Brooklyn New York USA; ^8^ Charles E. Schmidt College of Medicine, Florida Atlantic University Boca Raton Florida USA; ^9^ Department of Emergency Medicine Thomas Jefferson University/Jefferson Health Philadelphia Pennsylvania USA

## 1 Introduction

Artificial intelligence (AI) is rapidly changing the practice of medicine generally, and emergency medicine (EM) more specifically. Within healthcare, there is a rising demand for timely evidence discovery, evidence synthesis, and knowledge translation known as an evidence cycle [1, 2]. Our objective is to provide more efficient access to high‐quality evidence‐based medicine (EBM) recommendations in EM. The scope of this paper is the interaction between AI and evidence retrieval, appraisal, synthesis, translation and application rather than original (non‐systematic review with and without meta‐analysis) EBM generation.

## 2 Historical Evolution of Evidence Retrieval in Medicine

Modern evidence retrieval began with John Shaw Billings, a Civil War Surgeon, who found searching for medical literature to be both arduous and inefficient when researching surgical approaches to epilepsy [3]. By 1874, Billings enlisted military medical colleagues to design a systematic format for cataloging the literature, which later became the first Index Medicus (launched 1879) [3]. The 1873 cholera epidemic exposed the pressing need for comprehensive and accessible bibliographies. At the request of the US Surgeon General, Billings compiled a major index on the topic (cholera) in 1875 [3]. All of this would culminate in the founding of the National Library of Medicine (NLM), with its mission: to index the medical literature, provide it freely to others, thereby expanding access.

By 1875, the Index‐Catalogue (revised every 4 years) and Index Medicus (revised annually, through 2004) [4] created an unprecedented, structured route to medical writings. However, with steep increases in the volume of articles published, further innovation became necessary, giving rise to the Medical Subject Headings (MeSH) controlled vocabulary in 1960. MeSH standardized terminology, improved precision, and accelerated literature searches [5].

**FIGURE 1:**
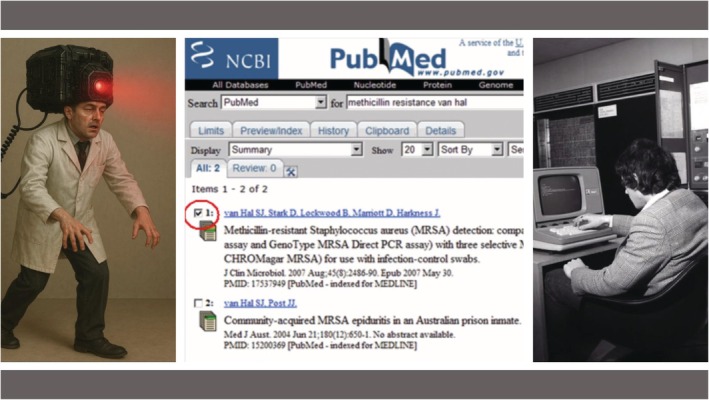
Timeline of evidence discovery tools.

A pivotal shift followed in 1966 with the creation of MEDLINE (Figure 1). Here, human indexers applied MeSH terms to articles and stored them in a retrieval‐ready database for librarians—and later, the public [6, 7]. The growth has remained dramatic: Krithara et al. report that in 2018 alone, over 1.3 million citations were added to MEDLINE/PubMed, a testament to both scholarly productivity and the rising burden on traditional indexing models [8]. To distribute this expanding information, condensed formats such as microfiche were introduced alongside traditional print.

Amid this era of exponential citation growth, an automatic semantic indexing was introduced in 2001. These systems aimed to transcend keyword search by leveraging machine learning and advanced natural language processing (NLP) to infer central concepts, assign high‐quality index terms, and offer more relevant search rankings [9]. The Medical Text Indexer (MTI) project exemplifies this, evolving from a tool for suggesting MeSH headings to human indexers to a system capable of mostly automated large‐scale indexing [8, 10].

Although the speed and scale of these systems honor Billings's initial drive for systematic access, challenges remain. Can machine‐generated indexing match the skeptical, evidence‐based appraisal and scrutiny of expert humans? Concerns about “hallucinated” citations and misinformation highlight these limitations. Nevertheless, algorithmic EBM AI techniques are opening new possibilities—not just for identifying information, but also for appraising the scientific literature [8].

## 3 Applications of AI in Evidence‐Based Practice

### 3.1 Evidence Retrieval

AI offers the potential opportunity to pose a clinical question in the hopes of receiving EBM response without having to do an exhaustive literature search and review of multiple studies. We tested several current AI options by posing a clinical question that was recently addressed by the American College of Emergency Physicians' (ACEP) Clinical Policy Committee. “In adult patients with a suspected acute ischemic stroke qualifying for intravenous thrombolysis, is tenecteplase safe and effective compared with alteplase?” [11].

The ACEP Clinical Policy Committee performed a systematic review on this critical question utilizing a predetermined strategy involving an extensive literature search. Relevant studies that were identified were graded on methodologic criteria and applicability to the clinical question. Articles meeting predetermined standards were the basis for the committee's evidence‐based recommendations. When this clinical question was posed to ChatGPT ver4.0, in less than 10 s, the following response appeared, presented in Table 2.

The Committee continued its work, and posed the same clinical question to Elicit. This AI chatbox was developed by a US non‐profit machine learning (ML) research laboratory. It uses AI large language models to identify relevant articles, synthesize data, and summarize information. There are other similar AI chatboxes designed to help summarize research literature (i.e., ResearchRabbit, Connected Papers, Scholarcy); however, we chose Elicit as an example. The response from Elicit, generated in approximately 10 min to generate, is also demonstrated in Table 2. The same inquiries were tested for reliability. The conclusion remained similar, though not identical.

As exemplified by the rapid responses of ChatGPT and Elicit to our clinical question, these AI tools offer the ability to pose clinical questions and rapidly receive evidence‐based responses that appear to be very similar to traditional methods of human‐derived evidence‐based information. It is noteworthy that poorly worded prompts can result in a different and potentially less accurate response.

However, little research has assessed the accuracy of the AI‐generated responses. Before AI is utilized to summarize the medical literature EBM recommendations, more research needs to occur to ensure accuracy and reproducibility.

## 4 Ethical, Educational, and Professional Considerations

### 4.1 Ethical Implications

As AI systems are increasingly used to enhance medicine, research, and medical education, the potential for legal liability emerges. While these tools can assist clinicians and trainees in synthesizing evidence rapidly, their integration may introduce legal and ethical challenges.

Emerging scholarship has highlighted both the pedagogical risks and unintended consequences of integrating generative AI into academic settings [12], including bias, hallucinations, data privacy concerns, and potential erosion of critical thinking [13]. These concerns are amplified by the rapid and widespread adoption of AI tools in education, with recent reports indicating that the majority of students now routinely use generative AI in their academic work [14]. In this context, unclear or absent institutional policies can lead to legal and ethical disputes. For example, a lawsuit filed by parents of a Massachusetts student following academic penalties for generative AI use, despite the absence of explicit policy guidance, illustrates the downstream consequences of inconsistent governance [15].

In addition to the legal implications, there are also ethical and moral implications of using AI in EBM. Medical professionalism is anchored in scholarly integrity, but LLMs lack moral agency or accountability [16, 17]. Because AI tools cannot have moral agency, they also cannot have legal accountability. Recent AI‐generated artifacts pose a novel threat. Investigations have revealed fabricated clinical trial data and hallucinating study citations produced by LLMs [18]. This can affect both novice students with classroom submissions or seasoned researchers submitting to academic journals alike.

To mitigate these emerging issues, formalized verification methods are essential. For example, mandated study pre‐registration, use of persistent digital identifiers (e.g., 2 factor identification), and transparent attribution of LLM contributions can reinforce investigator accountability. These frameworks help preserve the link between authorship and intellectual ownership while reducing the risk of AI‐generated misinformation infiltrating scholarly literature. In the educational sphere, explicit institutional policies must clarify how and when AI may be used in grading, course design, and learner assessment to support both faculty and learners. Learners must have clear expectations of when and how using AI is acceptable and not and if and how it should be acknowledged or cited.

Another concern in medical education is the over‐reliance on AI based solutions leading to a deficit in critical thinking. Indeed, recent data has shown that the use of AI to write an essay leads to reduced brain activity and connectivity compared to traditional methods [19]. Educators must balance the efficiency of utilizing AI to assist with writing and critical thinking tasks with the necessity to teach AI as a valuable tool to augment decision making and improve work flow. The potential for decreased cognitive performance from over reliance on AI tools by learners is a major dilemma. This is similar to the “calculator” debate in math education that occurred several decades ago and remains an issue to this day [20]. A recent scoping review noted that undergraduate medical education (UME) would benefit from AI becoming a mandated competency for medical students [21], and AI tool needing further evaluation for interaction with humans and dynamic competencies [22, 23]. Summarizing the information available and searching for it is part of the learning process that is now largely being omitted because of the rise of AI tools. We can't avoid AI at this point, so we need to teach our learners how to use it effectively and responsibly. The authors' review suggests that a “staged model”, with specific focus on training on the ethics, and inherent bias, of AI.

AI systems are inherently biased by their programming and the decisions that are made in establishing the logic utilized. One well‐known example is that ChatGPT would not say a racial slur to disarm a nuclear bomb to save millions of humans (because it was programmed it could never say that slur) [24]. AI models also retain biases in their training data sets. This is an especially important consideration in terms of systemic disparities like structural racism that have been engrained in medicine. Users must be mindful of not perpetuating these inequities and proactive in averting them when utilizing AI‐based tools.

Ultimately, safeguarding medical professionalism in the AI era depends on robust governance and shared values. To protect the rigor of EBM, it is essential to formalize verification standards and reinforce transparent attribution of LLM contributions. Institutions and journals must implement clear guidelines regarding AI use in manuscript preparation, literature synthesis, and critical appraisal. Similarly, educators must align AI policies in teaching with those of academic publishing, especially as learners rely increasingly on AI tools to engage with clinical literature. Educators must teach not only how to use AI, but also how to use it professionally and responsibly.

### 4.2 Patient‐Driven AI Use

In addition to physicians using AI to augment their work, doctors will also increasingly see patients who arrive with AI‐generated questions. Some of these patients will be highly informed while others will be misinformed due to lack of contextualization or by AI model generated misinformation (“hallucinations”). A pragmatic approach would be for physicians to acknowledge the patient's initiative and curiosity, while assessing the accuracy and relevance of the information. This would entail listening respectfully, clarifying misunderstandings, and addressing the misinformation or unrealistic expectations. An important part of this would be to explain the limitations of current AI‐generated answers, and to reinforce the value of personalized care, shared decision‐making, and professional judgment. A recent AMA column on “What Doctor's Wish Their Patients Knew” covered AI. It highlighted that AI is not always right, that AI can have bias, and that patients should learn to ask more discerning questions. (AMA) Physicians should be mindful that patients will likely come to them with some preconceived opinions about AI. A study from Yale found that patients have “positive views about AI's ability to improve care but had concerns about its potential for misdiagnosis, privacy breaches, reducing time with clinicians, and increasing costs, with racial and ethnic minority groups expressing greater concern.” [25]. That said, patients tend to view information from a human physician more positively than information which is AI generated [26].

## 5 Recommendations and Best Practices

AI has the potential to increase the practice of EBM by streamlining access to current literature, supporting clinical decision‐making, and enabling personalized recommendations at the point of care. However, to ensure AI tools complement rather than compromise the principles of EBM, clinicians and researchers must use them judiciously. The following recommendations outline best practices for integrating AI into evidence‐based practice while maintaining rigor, transparency, and clinician oversight.

1. **Maintain Human Oversight and Clinical Judgment**
  AI should augment, not replace, clinical reasoning. Physicians must critically appraise AI‐generated suggestions in the context of individual patient circumstances, values, and preferences. In addition, given the risk of hallucination, physicians should confirm references or practices with other resources.
2. **Prioritize Sites that Cite Sources (and then Evaluate Those Sources)**
  To reduce the risk of acting on AI hallucinations, it can be helpful to use systems that offer explainable outputs or cite specific evidence sources, enabling clinicians to understand how conclusions were reached and verify their validity. For example, Open Evidence provides references that can then be used to confirm the recommendations. Ultimately, it is the responsibility of the physician to vet these sources for accuracy. A lack of source evaluation may have played a role in the opioid pandemic [27].
3. **Hone Your Questions: Prompt Engineering**
  The quality of your question and phrasing can lead to better information from the AI platform. Some ChatGPT functions, such as deep research, will prompt the user for more information in order to narrow and hone the search. However, by providing more details, such as whether you are interested in pediatric or adult patients, or literature from only the last 5 or 10 years, or other parameters, will help you find the answers more easily. You can also use an AI platform to help you hone your question. You can put in your research question, and ask the AI to tell you what other parameters you should consider, or how you can narrow your search. Not only do they help get your answer more easily, prompt engineering is linked to reduction of hallucinations [28]. Using the traditional PICO format, below is an example of how to engage AI through “Prompt Engineering”

■Persona:

–Who are you, who your audience is, their voice your audience uses.

■Instruction/Input:

–What is the task you are asking AI to perform, what type of answer (notably the formatting) do you desire, how should AI handle atypical cases or data.

■Context/Clarity:

–In order to avoid hallucinations, place limits, request the response include positive and negative data (i.e., compare/contrast), request provide examples.

■Objective/Output:

–**What type of output you want**

4 **Use AI to Help with Information Synthesis**
  A function that AI does well is summarize and compare documents. For example, if you have downloaded 5–10 papers and want chat gpt or another AI platform to summarize, compare and contrast, or synthesize the information in the papers, you can request it. Or you can ask for brief summaries of the findings of each paper in order to decide which ones to read in greater detail.
5 Take a Course. Then Practice with Different Tools.

As above, while ChatGPT remains a default entry point for many users, it is important to recognize that alternative platforms may be better suited for specific academic and clinical tasks. Research‐focused tools such as Perplexity AI, Elicit, and Open Evidence are designed to support literature discovery, evidence synthesis, and clinical decision‐making.

Like any new technology, effective use of AI‐based tools requires deliberate practice and skill development. Modeling lifelong learning behaviors, clinicians and educators must be willing to adopt the role of learners. Numerous online resources and courses are available, with “prompt engineering” serving as a useful starting point to optimize interactions with AI systems. However, proficiency ultimately depends on iterative use across platforms.

Different tools offer distinct advantages (Table 1). For example, Elicit facilitates identification and synthesis of relevant research literature; Open Evidence supports evidence‐based responses to clinical questions (e.g., antibiotic selection); Perplexity AI integrates real‐time search with source attribution; and ChatGPT remains highly effective for summarization, synthesis, and drafting or revising written work. No single platform is optimal across all domains; therefore, developing fluency across multiple tools is essential for research, writing, and clinical practice.

**TABLE 1:** Comparison of common AI tools for research and clinical use.

Tool	Paid or free	Accuracy of evidence*	Other benefits
ChatGPT	Free (basic); Paid (Plus/Team)	Moderate–High	Writing, summarization, idea generation, coding support
Elicit	Free (limited); Paid tiers	High	Literature review automation, evidence tables, PICO querying
Research Rabbit	Free	Moderate	Visual citation mapping, discovery of related work
Consensus	Free (limited); Paid	High	Citation‐grounded answers, study synthesis
Microsoft Copilot	Free (limited); Paid	Moderate–High	Integration with Word/Excel/PowerPoint
Perplexity AI	Free; Paid (Pro)	High	Real‐time search with citations
Scite	Paid (limited free features)	High	Shows supporting vs. contradicting evidence
Open Evidence	Institutional access	High	Clinical decision support, guideline‐informed answers
Claude	Free (limited); Paid	Moderate–High	Long‐context reasoning, document analysis
Gemini	Free; Paid (Advanced)	Moderate–High	Google ecosystem integration, multimodal use

* Accuracy is context‐dependent and reflects general performance for academic and clinical tasks.

**TABLE 2:** Clinical policy, GPT, and Elicit output on the use of TNK and t‐PA for ischemic stroke.

ACEP clinical policy	Chat GPT	Elicit
ACEP Clinical Policy: “In adult patients with a suspected acute ischemic stroke qualifying for intravenous thrombolysis, is tenecteplase safe and effective compared with alteplase?” Patient Management Recommendations: Level A recommendations. None specified. Level B recommendations. Use either tenecteplase or alteplase in patients with acute ischemic stroke who qualify for thrombolysis. Level C recommendations. None specified. Potential Benefit of Implementing the Recommendations: Reduce errors in administration compared with alteplase. Improved short‐term neurologic outcomes. Improve the ease of patients needing to be transferred to a stroke facility. Improved 3‐month outcomes in patients with confirmed LVO. Potential Harm of Implementing the Recommendations: Incorrect dosing may increase the risk of complications.	Chat GPT: Your clinical question is clear and well‐structured. It focuses on a specific patient population, intervention, comparison, and outcomes — making it a solid PICO question: P: Adult patients with suspected acute ischemic stroke who qualify for intravenous thrombolysis, I: Tenecteplase, C: Alteplase, O: Safety and effectiveness (which may include functional outcomes, recanalization, symptomatic intracerebral hemorrhage, and mortality) Here's a concise summary of the current evidence (as of 2024) based on major trials and guidelines: Summary of Evidence: Tenecteplase vs. Alteplase in Acute Ischemic Stroke Key Trials: 1. EXTEND‐IA TNK (2018 & 2020) Population: Patients with large vessel occlusion eligible for thrombectomy Findings: Tenecteplase led to better reperfusion before thrombectomy and similar or better outcomes at 90 days compared to alteplase. 2. NOR‐TEST (2017 & 2022 follow‐up) Population: Broad stroke severity, including many with minor stroke or stroke mimics Findings: No significant difference in functional outcome; concerns about higher hemorrhage risk in more severe strokes in the NOR‐TEST 2 part. 3. ACT Trial (2022)/Large, multicenter RCT Findings: Tenecteplase was non‐inferior to alteplase for functional outcomes with no significant difference in safety (hemorrhage or mortality). Safety & Effectiveness Summary: Outcome:Tenecteplase vs. Alteplase Functional independence (mRS ≤ 2): Similar or slightly better with tenecteplase in some trials. Symptomatic intracerebral hemorrhage: Similar, though slightly variable depending on trial Mortality: No significant differenceEase of use:Tenecteplase is given as a single bolus, making it simpler to administer than the hour‐long alteplase infusion. Guidelines (as of 2024): AHA/ASA 2023: Tenecteplase (0.25 mg/kg) is recommended as an alternative to alteplase in patients with acute ischemic stroke who are eligible for thrombolysis, particularly when thrombectomy is planned. ESO/ESMINT: Support use of tenecteplase as an alternative in appropriate settings. Conclusion:Yes, tenecteplase appears to be a safe and effective alternative to alteplase for adult patients with acute ischemic stroke who are candidates for thrombolysis. It is non‐inferior in terms of functional outcomes and safety, and its ease of administration makes it attractive, especially in time‐critical settings. Would you like a summary in table form or formatted for a presentation or research project?	Elicit: Multiple studies show tenecteplase is generally as safe and effective as alteplase for acute ischemic stroke treatment, with most trials demonstrating similar outcomes but one noting increased risks. Abstract: Tenecteplase and alteplase yield largely similar functional and safety outcomes in adults with suspected acute ischemic stroke eligible for thrombolysis. In several trials using the modified Rankin Scale (mRS 0–1) at 90 days or 3 months, three studies reported non‐inferiority of tenecteplase to alteplase (e.g., one trial showed 36.9% versus 34.8% with a risk difference of 2.1% and a 95% confidence interval of −2.6 to 6.9; another reported a relative risk of 1.07 with a 95% confidence interval of 0.98–1.16), while one trial found tenecteplase to be superior (72% versus 40%, *p* = 0.02) and another found it inferior (odds ratio 0.45, 95% confidence interval 0.25–0.80, *p* = 0.0064). A meta‐analysis also noted a modest benefit for tenecteplase (relative risk 1.05, 95% confidence interval 1.01–1.10, *p* = 0.012). Safety findings across studies were similar in most cases. For example, one trial recorded symptomatic intracerebral hemorrhage in 3.4% of patients with tenecteplase and 3.2% with alteplase, and several studies noted comparable 90‐day or 3‐month mortality rates. One trial, however, reported higher rates of any intracerebral hemorrhage with tenecteplase (21% versus 7%; odds ratio 3.68, *p* = 0.0031) and an increased mortality rate at 3 months (16% versus 5%; odds ratio 3.56, *p* = 0.013). Other efficacy measures—such as early neurological improvement and penumbra salvage—demonstrated similar performance between the agents or favored tenecteplase to a modest degree. In summary, the papers reviewed indicate that, for patients with acute ischemic stroke treated within 4.5 h of symptom onset, tenecteplase produces functional outcomes that are generally comparable to those with alteplase and a similar safety profile, with the exception of one study noting increased hemorrhagic events and mortality with tenecteplase. Methods We analyzed 10 sources from an initial pool of 50, using 7 screening criteria. Each paper was reviewed for 6 key aspects that mattered most to the research question. The 10 sources cited by Elicit were not included here but can be found in the supplementary appendix

## 6 Future Directions

It is undoubtedly true that AI will have a significant impact on EM in the future, but the exact shape of this is yet to be seen. Some areas where there will likely be rapid growth include triage, diagnostic support, and operational efficiency. AI‐based tools can help prioritize high‐risk cases, such as sepsis or stroke, and predict patient disposition needs. For example, mobile‐based triage engines have shown early promise in improving ED throughput (Marchiori et al., 2020) [29]. As AI matures and is embedded into our electronic health records (EMRs), real time clinical decision support is likely. Decision‐support systems capable of synthesizing vitals, labs, and imaging data are emerging as tools to assist, but not replace clinical judgment [30].

Despite the promise, it is good to be skeptical. Many AI systems are *“black boxes”* with their reasoning opaque and hard to audit. This undermines clinician trust and makes it difficult to detect errors or embedded biases [31]. Furthermore, tools trained on historical datasets risk perpetuating existing health disparities. Generalizability (external validity) is another issue. Algorithms built for an urban/academic hospital population may not work in a rural/community setting. In addition, only a few AI models have undergone prospective validation in real‐world emergency care settings [30]. The so‐called *“AI chasm”—*or the gap between retrospective accuracy and clinical utility—remains wide.

Medico‐legal uncertainties should also temper our enthusiasm. If an AI tool makes a mistake, particularly one that leads to patient harm, who is accountable? As a result, many emergency physicians view AI tools as decision aids and not autonomous agents. A qualitative study found clinicians supported AI's potential but flagged risks of overreliance, reduced skill retention, and data privacy concerns [32].

To earn our trust moving forward, AI tools must be explainable, auditable, and governed by clear standards. Advances in *“explainable AI”* can help clarify model logic and allow clinicians to interrogate recommendations [31]. Regulators and professional societies must define performance thresholds, bias auditing practices, and accountability standards.

Integration of AI systems into clinical workflows is essential. They should interface seamlessly with EHRs and not be an additional barrier or burden to clinical tasks. Alerts must be accurate and actionable to avoid alarm fatigue. Maintaining physician oversight is critical. Clinicians must retain the authority to override AI outputs when they conflict with their clinical judgment. This “*human‐in‐the‐loop*” model protects patient safety and supports clinician autonomy.

AI also introduces a new reality for medical education. Future emergency physicians must be trained to appraise AI outputs critically. Curricula should incorporate foundational data literacy, principles of machine learning, and prompt engineering. UME needs to address the ethical concerns around the use of AI for both patient care and scholarship. To address concerns that automation may lead to skill atrophy, AI should be framed as an adjunct to clinical training [32].

The three pillars of EBM (best available evidence, clinical expertise, and patient values) require us to challenge AI outputs for their validity. Clinicians must verify AI‐suggested diagnoses or plans against guidelines and literature. They further require us to establish clear and transparent reporting guidelines when scholarship is disseminated as to how, when, and where in the process AI was utilized.

The road ahead calls for prospective studies to evaluate AI's real‐world impact on patient‐oriented outcomes, safety, and ED efficiency [30]. Implementation science can identify how best to embed AI into ED operations and mitigate unintended effects. Research in explainable and fair AI will help ensure future systems are transparent, ethical, and equitable.

## 7 Conclusion

In this dynamic era with new AI tools, we emphasize that humans are still accountable for errors in writing potentially caused by AI. Thus, we recommend the use of verification to check your work. Oftentimes, patients in the ED may have the same access to the AI tools that we do. It may be the only way to prevent ’hallucination’ that occurs with the routine use of LLMs in the clinical setting.

## Author Contributions

**Joshua Davis:** conceptualization, writing – original draft, writing – review and editing. **Martin Wegman:** conceptualization, writing – original draft. **Richard D. Shih:** conceptualization, writing – original draft, writing – review and editing. **Lars K. Beattie:** conceptualization, writing – original draft. **Laura Melville:** conceptualization, writing – original draft. **Sangil Lee:** conceptualization, methodology, writing – original draft, writing – review and editing. **Christina Shenvi:** conceptualization, writing – original draft, supervision. **Ken Milne:** conceptualization, writing – original draft. **Bryan Kane:** conceptualization, writing – original draft, writing – review and editing, supervision.

## Funding

The authors have nothing to report.

## Ethics Statement

The authors have nothing to report.

## Conflicts of Interest

The authors declare no conflicts of interest.

## Data Availability

The data that support the findings of this study are available on request from the corresponding author. The data are not publicly available due to privacy or ethical restrictions.

## References

[1] R. F. Heller , A. Verma , I. Gemmell , R. Harrison , J. Hart , and R. Edwards , “Critical Appraisal for Public Health: A New Checklist,” Public Health 122, no. 1 (2008): 92–98, 10.1016/j.puhe.2007.04.012. 17765937

[2] S. Tenny and M. A. Varacallo , Evidence‐Based Medicine (StatPearls. StatPearls Publishing, 2025). 29262040

[3] J. A. Curran , “BILLINGS at Gettysburg,” New England Journal of Medicine 269 (1963): 23–27, 10.1056/NEJM196307042690106. 14024307

[4] W. Miles , A History of the National Library of Medicine ‐ Google Books (Google Books, 1982), https://www.google.com/books/edition/A_History_of_the_National_Library_of_Med/e8SPvZX_rE0C?hl=en&gbpv=0.

[5] F. B. Rogers , “Letters to the Editor,” in Communications to the Editor, vol. 40 (Medical Library Association, 1963), 228.

[6] D. A. Lindberg and H. M. Schoolman , “The National Library of Medicine and Medical Informatics,” Western Journal of Medicine 145, no. 6 (1986): 786–790. 3544508 PMC1307151

[7] C. B. Lipscomb , Medical Subject Headings (MeSH) (Medical Library Association, 2000). PMC35238 10928714

[8] A. Krithara , J. G. Mork , A. Nentidis , and G. Paliouras , “The Road From Manual to Automatic Semantic Indexing of Biomedical Literature: A 10 Years Journey,” Frontiers in Research Metrics and Analytics 8 (2023): 1250930, 10.3389/frma.2023.1250930. 37841902 PMC10576528

[9] A. R. Aronson , “Effective Mapping of Biomedical Text to the UMLS Metathesaurus: The Meta Map Program,” AMIA Annual Symposium Proceedings (2001): 17–21. PMC2243666 11825149

[10] J. Mork , A. Aronson , and D. Demner‐Fushman , “12 Years On‐Is the NLM Medical Text Indexer Still Useful and Relevant?,” Journal of Biomedical Semantics 8, no. 1 (2017): 8, 10.1186/s13326-017-0113-5. 28231809 PMC5324252

[11] American College of Emergency Physicians Clinical Policies Subcommittee (Writing Committee) on Acute Ischemic Stroke , B. M. Lo , and C. R. Carpenter , “Clinical Policy: Critical Issues in the Management of Adult Patients Presenting to the Emergency Department With Acute Ischemic Stroke,” Annals of Emergency Medicine 82, no. 2 (2023): e17–e64, 10.1016/j.annemergmed.2023.03.007. 37479410

[12] Plagiarism Today , “The Backlash Against AI Accusations,” (2025), https://www.plagiarismtoday.com/2025/01/23/the‐backlash‐against‐ai‐accusations/.

[13] S. M. Hosseini , “AI Misuse and Passiveness of Students in Medical Education,” Advances in Physiology Education 49, no. 4 (2025): 1009–1013, 10.1152/advan.00164.2025. 40938814

[14] C. Ganjavi , M. Eppler , D. O’Brien , et al., “ChatGPT and Large Language Models (LLMs) Awareness and Use. A Prospective Cross‐Sectional Survey of U.S. Medical Students. PLOS Digit,” Health 3, no. 9 (2024): e0000596, 10.1371/journal.pdig.0000596. PMC11376538 39236008

[15] Blog : AI‐Lawsuit‐Challenges‐School‐Rules , “Blog : AI‐Lawsuit‐Challenges‐School‐Rules,” (2025), https://www.gross‐shuman.com/blog/ai‐lawsuit‐challenges‐school‐rules.

[16] C. Véliz , “Moral Zombies: Why Algorithms Are Not Moral Agents,” AI & SOCIETY 36 (2021): 487–497, 10.1007/s00146-021-01189-x. 36568029 PMC7613994

[17] M. Zafar , “Normativity and AI Moral Agency,” AI and Ethics 5, no. 3 (2025): 2605–2622, 10.1007/s43681-024-00566-8.

[18] F. Aljamaan , M.‐H. Temsah , I. Altamimi , et al., “Reference Hallucination Score for Medical Artificial Intelligence Chatbots: Development and Usability Study,” JMIR Medical Informatics 12 (2024): e54345, 10.2196/54345. 39083799 PMC11325115

[19] N. Kosmyna , E. Hauptmann , Y. T. Yuan , et al., “Your Brain on ChatGPT: Accumulation of Cognitive Debt When Using an AI Assistant for Essay Writing Task,” arXiv (2025), 10.48550/arxiv.2506.08872.

[20] J. Monaghan , “The Calculator Debate,” in Tools and Mathematics. Vol 110. Mathematics Education Library (Springer International Publishing, 2016), 305–331, 10.1007/978-3-319-02396-0_13.

[21] E. H. H. Rincón , D. Jimenez , L. A. C. Aguilar , J. M. P. Flórez , Á. E. R. Tapia , and C. L. J. Peñuela , “Mapping the Use of Artificial Intelligence in Medical Education: A Scoping Review,” BMC Medical Education 25, no. 1 (2025): 526, 10.1186/s12909-025-07089-8. 40221725 PMC11993958

[22] C. Preiksaitis and C. Rose , “Opportunities, Challenges, and Future Directions of Generative Artificial Intelligence in Medical Education: Scoping Review,” JMIR Medical Education 9 (2023): e48785, 10.2196/48785. 37862079 PMC10625095

[23] M. M. Triola and A. Rodman , “Integrating Generative Artificial Intelligence Into Medical Education: Curriculum, Policy, and Governance Strategies,” Academic Medicine 100, no. 4 (2025): 413–418, 10.1097/ACM.0000000000005963. 39705530

[24] “ChatGPT Goes Viral for Choosing Global Destruction Over Using Racial Slur,” https://thenationaldesk.com/news/americas‐news‐now/chatgpt‐goes‐viral‐for‐choosing‐global‐destruction‐over‐using‐racial‐slur‐openai‐artificial‐intelligence‐chat‐bot‐chatbot‐google.

[25] D. Khullar , L. P. Casalino , Y. Qian , Y. Lu , H. M. Krumholz , and S. Aneja , “Perspectives of Patients About Artificial Intelligence in Health Care,” JAMA Network Open 5, no. 5 (2022): e2210309, 10.1001/jamanetworkopen.2022.10309. 35507346 PMC9069257

[26] M. Reis , F. Reis , and W. Kunde , “Influence of Believed AI Involvement on the Perception of Digital Medical Advice,” Nature Medicine 30, no. 11 (2024): 3098–3100, 10.1038/s41591-024-03180-7. PMC11564086 39054373

[27] P. T. M. Leung , E. M. Macdonald , M. B. Stanbrook , I. A. Dhalla , and D. N. Juurlink , “A 1980 Letter on the Risk of Opioid Addiction,” New England Journal of Medicine 376, no. 22 (2017): 2194–2195, 10.1056/NEJMc1700150. 28564561

[28] S. Nishisako , T. Higashi , and F. Wakao , “Reducing Hallucinations and Trade‐Offs in Responses in Generative AI Chatbots for Cancer Information: Development and Evaluation Study,” JMIR Cancer 11 (2025): e70176, 10.2196/70176. 40934488 PMC12425422

[29] C. Marchiori , D. Dykeman , I. Girardi , et al., “Artificial Intelligence Decision Support for Medical Triage,” American Medical Informatics Association Annual Symposium Proceedings 25 (2020): 793–802. PMC8075483 33936454

[30] H. Kareemi , K. Yadav , C. Price , et al., “Artificial Intelligence‐Based Clinical Decision Support in the Emergency Department: A Scoping Review,” Academic Emergency Medicine 32, no. 4 (2025): 386–395, 10.1111/acem.15099. 39905631

[31] A. Rajaram , H. Li , J. K. Holodinsky , et al., “Opening the Black Box: Challenges and Opportunities Regarding Interpretability of Artificial Intelligence in Emergency Medicine,” Canadian Journal of Emergency Medicine 27, no. 2 (2025): 83–86, 10.1007/s43678-024-00827-9. 39962037

[32] J. Stewart , S. Freeman , E. Eroglu , et al., “Attitudes Towards Artificial Intelligence in Emergency Medicine,” Emergency Medicine Australasia 36, no. 2 (2024): 252–265, 10.1111/1742-6723.14345. 38044755

